# Identification of key candidate genes for ovarian cancer using integrated statistical and machine learning approaches

**DOI:** 10.1093/bib/bbaf602

**Published:** 2025-12-17

**Authors:** Md Ali Hossain, Tania Akter Asa, Md Shofiqul Islam, Mohammad Zahidur Rahman, Mohammad Ali Moni

**Affiliations:** NanoBio Technology Center, and Computer Science and Engineering, Daffodil International University, Birulia, Savar, Dhaka-1216, Bangladesh; Computer Science and Engineering, Jahangirnagar University, Savar, Dhaka-1342, Bangladesh; NanoBio Technology Center, and Computer Science and Engineering, Daffodil International University, Birulia, Savar, Dhaka-1216, Bangladesh; Computer Science and Engineering, Jagannath University, 9-10 Chittaranjan Avenue, Sadarghat, Dhaka-1100, Bangladesh; Institute for Intelligent Systems Research and Innovation (ISSRI), Deakin University, 75 Pigdons Road, 3216 Warun Ponds, Victoria, Australia; Computer Science and Engineering, Jahangirnagar University, Savar, Dhaka-1342, Bangladesh; Health Sciences Research Center (HSRC), Deanship of Scientific Research, Imam Mohammad Ibn Saud Islamic University (IMSIU), Riyadh, Saudi Arabia; AI and Digital Health Technology, Rural Health Research Institute, Charles Sturt University, Orange, 2800 NSW, Australia; AI and Digital Health Technology, AI and Cyber Futures Institute, Charles Sturt University, Bathurst, 2795 NSW, Australia

**Keywords:** ovarian cancer (OC), support vector machine (SVM), protein–protein interaction (PPI), meta-hub genes, WGCNA analysis, survival analysis

## Abstract

Ovarian cancer (OC) is a highly lethal malignancy worldwide, necessitating the identification of key genes to uncover its molecular mechanisms and improve diagnostic and therapeutic strategies. This study utilized statistical and machine learning approaches to identify key candidate genes for OC. Three microarray datasets were obtained from the gene expression omnibus database, and analysis began with normalization and differential gene expression analysis using the Limma package. Highly discriminative differentially expressed genes (HDDEGs) were identified through a support vector machine-based approach, yielding 84 overlapping HDDEGs across the datasets. Enrichment analysis of HDDEGs was conducted using DAVID. A protein–protein interaction network constructed via STRING pinpointed central hub genes using CytoHubba metrics. Significant modules were analyzed with molecular complex detection, identifying 18 central hub genes, 11 hub module genes, and 54 meta-hub genes. The intersection of these three gene sets revealed eight shared key genes (FANCD2, BUB1B, BUB1, KIF4A, DTL, NCAPG, KIF20A, and UBE2C). Weighted gene co-expression network analysis identified key modules linked to clinical traits and confirmed grouping eight key candidate genes into a single cluster. These genes were validated using two independent datasets (GSE38666 and TCGA-OC), with area under the curve and survival analyses underscoring their predictive and prognostic significance in OC. This integrative approach advances understanding of OC’s molecular basis, identifies potential biomarkers, and emphasizes the clinical relevance of the eight key candidate genes for OC diagnosis, prognosis, and treatment.

## Introduction

Ovarian cancer (OC) is the fifth leading cause of cancer-related deaths among women in developed nations and the eighth most prevalent cancer worldwide, characterized by high mortality rates primarily due to late-stage detection [1, 2]. The 5-year survival rate is below 45%, mainly because most cases are diagnosed at advanced stages when symptoms are vague or nonspecific [2]. The key risk factors include advanced age, genetic mutations (e.g. BRCA1 and BRCA2), family history, hormone replacement therapy, and nulliparity [1, 3]. Reproductive factors, including late menopause and infertility, contribute to an increased risk, while pregnancy and the use of oral contraceptives are protective [3, 4]. Lifestyle factors, including obesity, high-fat diets, and smoking, also contribute to the risk, alongside environmental exposures like talc and pesticides, though the latter requires more research [3]. OC’s etiology remains complex, with different histological subtypes potentially having distinct risk profiles [5]. Despite advances in treatment, including surgery and chemotherapy, up to 75% of patients develop chemoresistance, resulting in poor outcomes [6]. The identification of significant genes could enhance early detection and improve treatment options, addressing the urgent need for better diagnostic tools and therapeutic approaches. However, the absence of effective early detection methods, genetic diagnostics, and adequate treatment facilities continues to drive the rise in cancer-related deaths [2, 6]. As a result, improving gene-based detection and expanding treatment capabilities are critical to reducing mortality rates associated with OC.

In recent years, bioinformatics analysis [7] has emerged as a powerful tool for unraveling the molecular landscape of OC, aiding in finding important prognostic genes and molecular pathways. Numerous studies have identified potential biomarkers and core genes associated with OC progression, metastasis, and treatment response [8–46]. Zhao *et al.* [8] identified six hub genes (AURKA, BUB1B, CENPF, KIF11, KIF23, and TOP2A) associated with poor prognosis in OC through bioinformatics and experimental validation. Su *et al.* [9] identified 22 hub genes strongly linked to poor OC survival through bioinformatics analysis, while in their other work [10], they identified seven immune-related hub genes using weighted gene co-expression network analysis (WGCNA), highlighting LY9 and SLAMF1 as key regulators in a ceRNA network, thereby providing insights into potential immunotherapy targets. Furthermore, a study by Li *et al.* [11] discovered four hub genes—COL4A1, SDC1, CDKN2A, and TOP2A—correlating with the overall survival of OC.

These studies have illuminated the complex molecular mechanisms driving ovarian tumorigenesis, laying the foundation for advancing targeted therapies and personalized treatment strategies. In OC research, many studies focus on identifying key genes using protein–protein interaction (PPI) networks [8–27, 29–47]. A major challenge is finding relevant biomarkers or genes that provide insight into disease mechanisms. Machine learning (ML) techniques have gained attention for addressing this issue by analyzing complex genetic data [48–52]. Despite efforts to identify potential candidate genes for OC, the task remains difficult. More research is needed to improve gene identification and understand the molecular mechanisms driving the development and progression of the disease. This is essential for advancing diagnostics and treatment strategies.

This study aimed to identify key candidate genes associated with OC by integrating bioinformatics and ML methodologies. Using three microarray gene expression (MGE) datasets, we systematically identified differentially expressed genes (DEGs) and selected highly discriminative DEGs (HDDEGs) with strong classification performance. Functional enrichment analysis, PPI network construction, and hub gene identification were performed to uncover crucial molecular players. Furthermore, module analysis, meta-hub gene (MHG) integration, and WGCNA were utilized to refine our gene selection. Finally, we validated the prognostic relevance of these key candidate genes using independent datasets and survival analysis. Our findings provide novel insights into the molecular mechanisms underlying OC and can potentially contribute to the development of targeted therapies and precision medicine strategies.


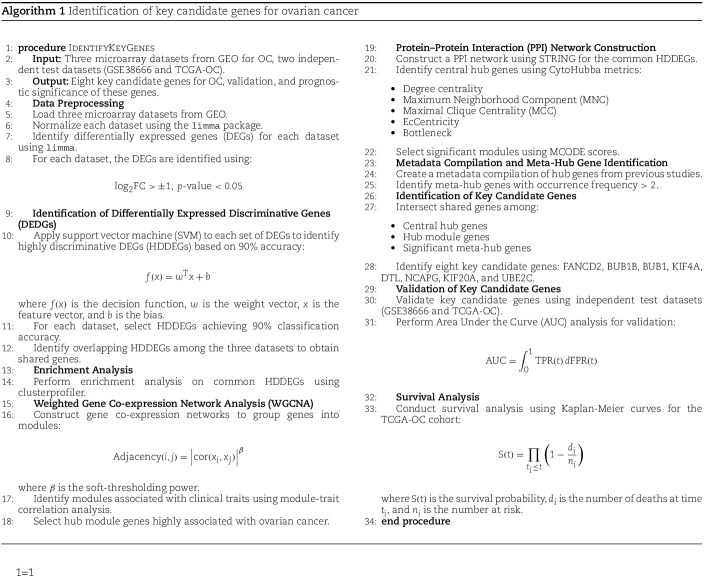


## Materials and methods

**Figure 1:**
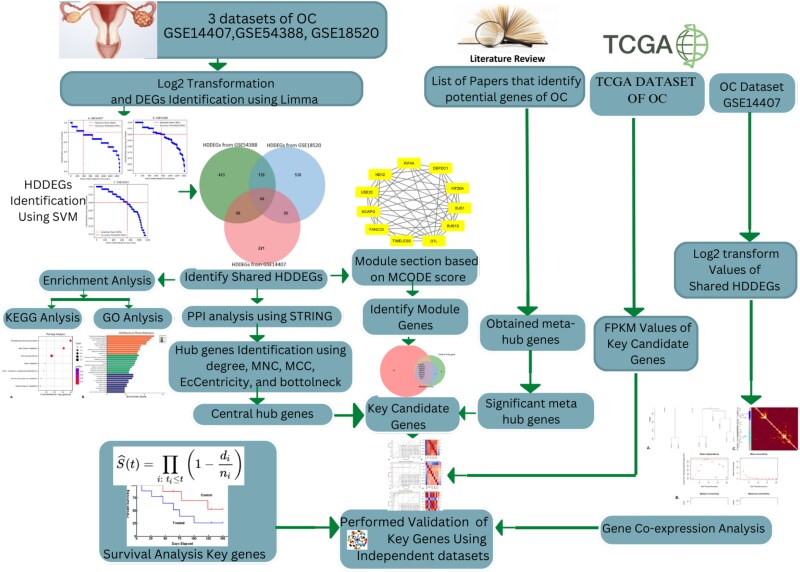
Workflow of the study: The analysis starts with the collection of transcriptomic datasets and significant gene metadata from the literature. Data preprocessing ensures normalization, followed by identifying DEGs using the Limma package. Subsequently, DEGs are filtered using an SVM-based approach to identify 84 overlapping HDDEGs. Enrichment analysis and PPI network construction are performed, and five analytical approaches (connectivity degree, MNC, MCC, EcCentricity, and bottleneck) are applied to identify 18 central hub genes. Additionally, 11 hub module genes and 54 MHGs pivotal to cancer biology are identified. By overlapping these three gene sets, eight shared key genes (FANCD2, BUB1, BUB1B, KIF4A, DTL, NCAPG, KIF20A, and UBE2C) are highlighted. WGCNA confirms the grouping of these eight key genes into a single cluster. Validation through AUC and survival analyses underscores their predictive and prognostic significance in OC. This workflow integrates bioinformatics and ML for robust biomarker discovery.

In this research, we used microarray data of OC from the publicly available NCBI Gene Expression Omnibus (GEO) database (http://www.ncbi.nlm.nih.gov/geo/). Additionally, we incorporated mRNA-seq data of OC from The Cancer Genome Atlas (TCGA) through the TCGA genome data analysis center (http://gdac.broadinstitute.org/). Figure 1 illustrates the detailed workflow of this study, outlining the sequential steps from data acquisition to key gene identification and validation. Algorithm 1 presents the algorithm proposed in this study, highlighting the computational approaches used for feature selection, classification, and survival analysis.

First, we selected DEGs from each of the three datasets. Then, we applied an SVM with a radial basis function (RBF) kernel to the DEGs from each dataset to calculate the classification accuracy for each gene. DEGs that achieved a classification accuracy of >90.0% were selected. We then identified the overlapping DEGs among the three datasets, which we termed differentially expressed discriminative genes (HDEDGs). Next, we performed enrichment analysis on the common DEDGs using the DAVID tool. We constructed PPI networks using STRING and visualized them with Cytoscape. Using the cytoHubba plugin, hub genes were found using a variety of metrics, including degree, maximal neighborhood component (MNC), maximal clique centrality (MCC), proximity, and bottleneck. Genes that were consistently identified across all of these measures were referred to as central hub genes. We identified essential modules and the genes that accompany them by using molecular complex detection (MCODE) for cluster or module analysis to further hone our findings. Important MHGs were also taken from previous research. Key or core candidate genes were found by overlapping the central hub genes, possible module hub genes, and important MHGs, proving their capacity to distinguish OC patients from healthy controls. Using two separate test datasets, we confirmed the significant discriminative potential of candidate genes. We also performed survival analysis for the identified major candidate genes in OC patients.

## Algorithm: identification of key candidate genes for ovarian cancer

Algorithm 1 provides the overall workflow process of this study, outlining the systematic approach for identifying and validating key biomarkers in OC. The algorithm begins with data collection and preprocessing, followed by normalization and differential expression analysis using Limma. ML methods, such as SVM, filter the DEGs to derive 84 overlapping HDDEGs. These genes undergo enrichment analysis and PPI network construction, leading to the identification of 18 central hub genes, 11 hub module genes, and 54 MHGs pivotal to cancer biology. A Venn diagram overlaps these gene sets to highlight eight shared key genes (FANCD2, BUB1, BUB1B, KIF4A, DTL, NCAPG, KIF20A, and UBE2C), which are further confirmed through WGCNA clustering. Validation using AUC and survival analyses underscores the clinical relevance of these findings. This process ensures a comprehensive and integrative approach to biomarker discovery in OC.

### Materials

Three publicly available MGE datasets, GSE18520, GSE54388, and GSE14407, were employed in this study to identify potential key genes linked to OC progression. To further validate the significance of these genes, two additional independent datasets—GSE38666 from the GEO database and TCGA-OC (Ovarian Serous CystadenoCarcinoma, Nature 2011) from The Cancer Genome Atlas (TCGA)—were used. These datasets offer crucial gene expression data that enable a more comprehensive analysis. The microarray datasets were sourced from the GEO repository, while the TCGA data were retrieved through the TCGA portal. We also included normal ovarian tissue data comprising 180 patient samples obtained from the Genotype-Tissue Expression (GTEx) project, accessible at https://www.gtexportal.org/home/. To maintain consistency in the analysis, all NCBI datasets underwent log2 transformation and quintile normalization, while the TCGA dataset was normalized using the fragments per kilobase of transcript per million mapped reads (FPKM) method. Even though these datasets are openly accessible, all steps in their processing were strictly adhered to the relevant ethical guidelines and regulatory standards for research involving human genetic data. The inclusion criteria for this study were based on publicly available transcriptomic datasets containing both normal and OC samples. OC datasets were selected based on histopathological confirmation, microarray/RNA-seq data availability, and well-annotated clinical information. While the datasets included tumor stages I–IV, our analysis focused on distinguishing normal samples from OC samples to ensure a precise and robust classification. This approach allows for a more straightforward comparison of gene expression differences between healthy and diseased states, aiding in identifying potential biomarkers relevant to OC diagnosis and prognosis. Table 1 provides a detailed overview of the datasets used.

**Table 1:** Summary of total, normal, and OC samples across different datasets

**Dataset**	**Total samples**	**Normal**	**Ovarian cancer**
GSE14407	24	12	12
GSE54388	14	6	8
GSE18520	63	10	53
GSE38666	45	20	25
TCGA-OC	559	180	379

### Methods

This study aimed to find key candidate genes for OC by analyzing three MGE datasets. Our approach involved several steps to ensure the robust identification of these genes. The following sections describe the general process of our suggested methodology for identifying essential candidate genes for OC. Details of the methodological approaches are provided in the following sections.

#### Differentially expressed genes identification

DEGs between OC and healthy controls were identified by analyzing each dataset with the “limma” package in R (v4.1.2), applying a significance threshold of adj. $P$-value $<.05$ and log2FC $>\pm 1$. Probes were mapped to gene symbols using the “Bioconductor annotation” package, with the lowest adj. $P$-value chosen for multiple probe matches. Visualizations included a volcano plot (via “ggplot2”) and a heatmap of DEG expression (via “NMF”).

### Highly discriminative differentially expressed genes identification in ovarian cancer datasets

Finding a hyperplane in a high-dimensional space that successfully separates OC survivors from healthy people utilizing the following discriminant algorithm is the main objective of SVM: 


(6) \begin{align*}& f(x) = w^{T} x + b\end{align*}

where $b$ indicates bias value.

In this study, we employed an RBF kernel, which is mathematically expressed as: 


(7) \begin{align*}& K(x, y) = \exp(-\gamma ||x - y||^{2})\end{align*}

We adjusted the cost parameter $C$ and the gamma parameter $\gamma $ using a grid search to optimize classification performance. To enhance accuracy, SVM was used as a gene selection technique, and the process is outlined below:

1. Select one gene from the identified DEGs.
2. Train an SVM model with five-fold CV for that gene.
3. Calculate the classification accuracy.
4. Repeat for all DEGs.
5. Rank genes by classification accuracy.
6. Select genes with accuracy above 90%.

## Identification of shared highly discriminative differentially expressed genes

After identifying HDDEGs using SVM, we determined the common HDDEGs across the three datasets. This can be expressed mathematically as follows: 


\begin{align*} & \text{Common HDDEGs} = \bigcap_{i=1}^{r} \text{HDDEGs}_{i} \end{align*}

where $ r $ represents the number of GEO datasets analyzed (in this case, $ r = 3 $).

### Analyzing the enrichment of shared highly discriminative differentially expressed genes of ovarian cancer

We used the clusterProfiler program to do enrichment analysis on the HDDEGs that were found in order better to understand the etiology and course of OC. The Kyoto Encyclopedia of Genes and Genomes (KEGG) and Gene Ontology (GO) pathways were incorporated into this investigation. A $P$-value < $.05$ was established as the significance criterion to identify significant relationships.

#### Construction of protein–protein interaction Network and Identification of central hub gene

The potential interactions among common genes HDDEGs obtained from three datasets were predicted using STRING version 11.5 (www.string-db.org). Only interactions with a confidence score of $\geq $0.4 and the complete STRING network were considered to construct a PPI network, while limiting the number of interactors to 0. STRING integrates multiple interaction sources, including literature, co-expression, gene fusion, and neighborhood-based associations. Cytoscape version 3.9.1 visualizes the network in the generated PPI data.

We computed necessary network measures, such as the degree of connectivity, MNC, MCC, and the centralities of EcCentricity and bottleneck, using the cytoHubba plugin to find hub genes. The top 20 genes from each technique were chosen as possible hub genes after these metrics were ordered in descending order. Considering the overlap of hub genes derived from all five metrics (degree of connection, MNC, MCC, proximity, and bottleneck), which can be expressed mathematically as follows, central hub genes were found: 


\begin{align*} & \text{Central Hub Genes} = \bigcap_{i=1}^{hg} H_{i} \end{align*}

where $ hg = 5 $ represents the number of hub gene identification methods used.

#### Hub modules and their associated genes identification

To identify the PPI network’s most tightly linked modules, we applied the MCODE algorithm. Node degree $\geq 2$, haircut option activated for clustering, node score cutoff set at 0.2, K-core set at 2, and a maximum depth of 100 were the thresholds used to identify the modules. The number of nodes in each module, as well as their MCODE score, was used to choose potential modules. The following formula was used to identify the genes present in the hub modules: 


\begin{align*} & \text{Hub Module Genes} = \sum_{i=1}^{m} G_{i} \end{align*}

where $m$ denotes the total count of significant modules identified.

#### Identification of significant meta-hub genes from comprehensive metadata analysis

Let $ G_{i} $ represent the set of hub genes identified in the $ i $th study, where $ i = 1, 2, \dots , 40 $.

The $ MHG $ list is the union of all hub gene sets from each of the 40 studies: 


\begin{align*} & MHG = \bigcup_{i=1}^{40} G_{i} \end{align*}

This represents the collection of all unique hub genes identified across the 40 studies.

If we define $ f(g) $ as the number of studies in which each gene $ g $ is identified as a hub gene, then the set of significant meta-hub genes (SMHG)—that is, the genes appearing in more than one study—can be defined as: 


\begin{align*} & SMHG = \left\{ g \in MHG \mid f(g) \geq 2 \right\} \end{align*}

#### Identification of key candidate genes


\begin{align*} & \text{Key Candidate Genes} = \bigcap_{i=1}^{k} G_{i} \end{align*}

Where $ G_{i} $ represents the gene sets from each identification method (e.g. central hub genes, hub module genes, and significant meta-hub genes). $ k $ is the number of gene identification methods (in this case, $ k = 3 $).

#### Validation of key candidate genes

##### Assessing discriminative power via receiver operating characteristic curve analysis

We employed two separate datasets, TCGA-OC from the TCGA database and GSE38666 from the GEO database, to validate the critical candidate genes found in our investigation. Table 1 provides more details regarding these datasets. As part of the validation phase, the AUC using receiver operating characteristic (ROC) curve analysis was used to evaluate the discriminative potential of the chosen genes.

We used a leave-one-out cross-validation (CV) technique to execute logistic regression after selecting each gene and its associated class label for the ROC analysis. Python was utilized to calculate the AUC values. To do logistic regression, create ROC curves, and determine AUC values, we used Python’s scikit-learn module. We contrasted the performance of these distinct test datasets with that of GSE18520, one of our training datasets, to further evaluate the robustness of the chosen key candidate genes.

#### Survival analysis of key candidate genes of ovarian cancer

The Kaplan–Meier (KM) Plotter program was used to create KM survival curves in order to evaluate the possible impact of the identified key candidate genes for the survival of patients with OC https://kmplot.com/analysis [53]. This analysis focused on evaluating the prognostic significance of these key OC genes, offering insights into their impact on patient outcomes and their potential as biomarkers for disease progression.

#### Weighted gene co-expression network analysis analysis of common highly discriminative differentially expressed genes

We used WGCNA to investigate the co-expression patterns of HDDEGs shared across three datasets (GSE18520, GSE54388, and GSE14407). Among these, we considered GSE14407 after normalization for further analysis. This approach enabled us to identify gene modules with high co-expression, potentially revealing key biological pathways relevant to OC.

Using the WGCNA package in R, a soft-thresholding power was chosen to ensure scale-free topology, followed by hierarchical clustering to group genes into distinct modules. Module-trait relationships were assessed to determine significant associations between modules and clinical traits or disease phenotypes. The hub genes within these key modules were identified based on their intra-modular connectivity, providing further insights into the functional relevance of the common HDDEGs in OC.

## Result

### Differentially expressed genes identification

To find DEGs from each of the three GEO datasets (**GSE14407**, **GSE54388**, and **GSE18520**), we utilized the *limma* package, applying the statistical criteria outlined in the Methods section. we found:

- **GSE14407**: a total of **1486 DEGs**, with **661 upregulated** and **825 downregulated** genes.
- **GSE54388**: a total of **1415 DEGs**, with **695 upregulated** and **720 downregulated** genes.
- **GSE18520**: a total of **1143 DEGs**, with **629 upregulated** and **514 downregulated** genes.

**Figure 2:**
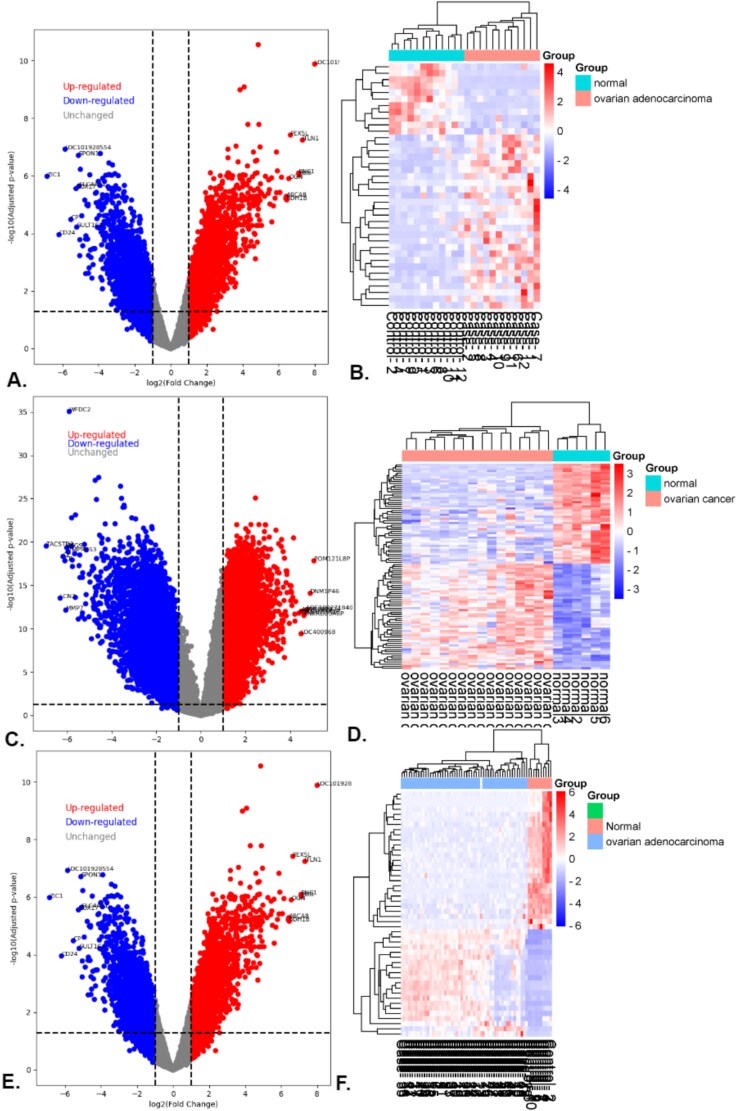
Visualization of differential gene expression and clustering: (A) Volcano plot and (B) heatmap for GSE14407; (C) volcano plot and (D) heatmap for GSE54388; (E) volcano plot, and (F) heatmap for GSE18520.

The volcano plots and heatmaps of these DEGs are presented in Fig. 2.

#### Identification of shared highly discriminative differentially expressed genes

The detected DEGs from each of the three datasets (**1486 DEGs for GSE14407**, **1415 DEGs for GSE54388**, and **1143 DEGs for GSE18520**) were subjected to an application of the SVM using an RBF kernel to identify the HDDEGs for OC patients. The classification accuracy for each gene was computed based on the procedure outlined in the methodology section.

**Figure 3:**
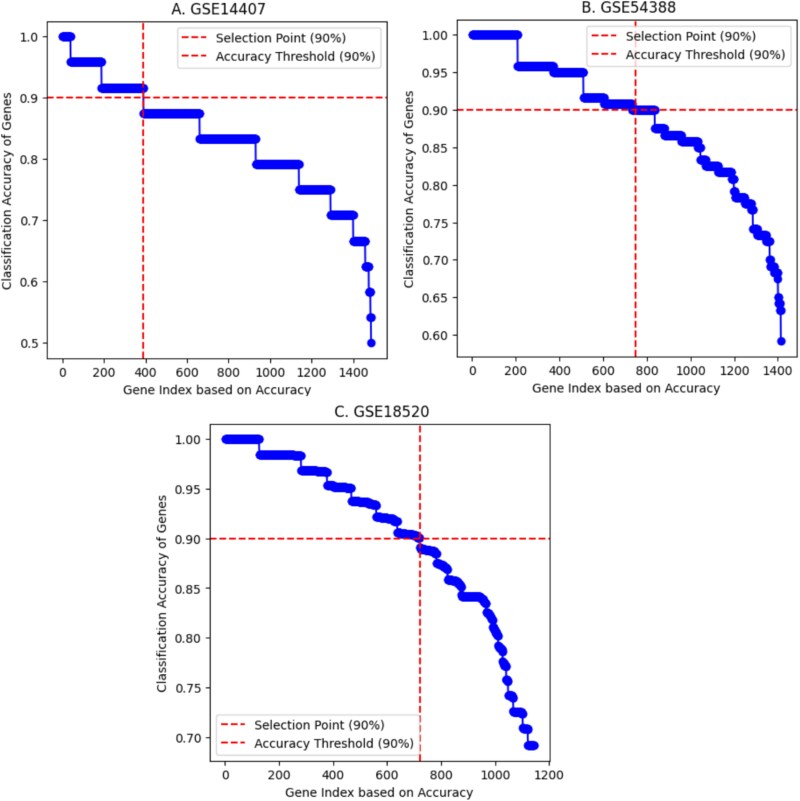
Evaluating gene classification accuracy with SVM across three GEO datasets: (A) GSE18520, (B) GSE54388, and (C) GSE14407.

We calculated the classification accuracies for each dataset for all DEGs and ordered them in descending order. After applying SVM, **390 DEGs from GSE14407**, **712 DEGs from GSE54388**, and **811 DEGs from GSE18520** were selected as HDDEGs, with an accuracy of 90.0%. The detailed classification accuracy distributions for individual datasets are presented in Fig. 3.

**Figure 4:**
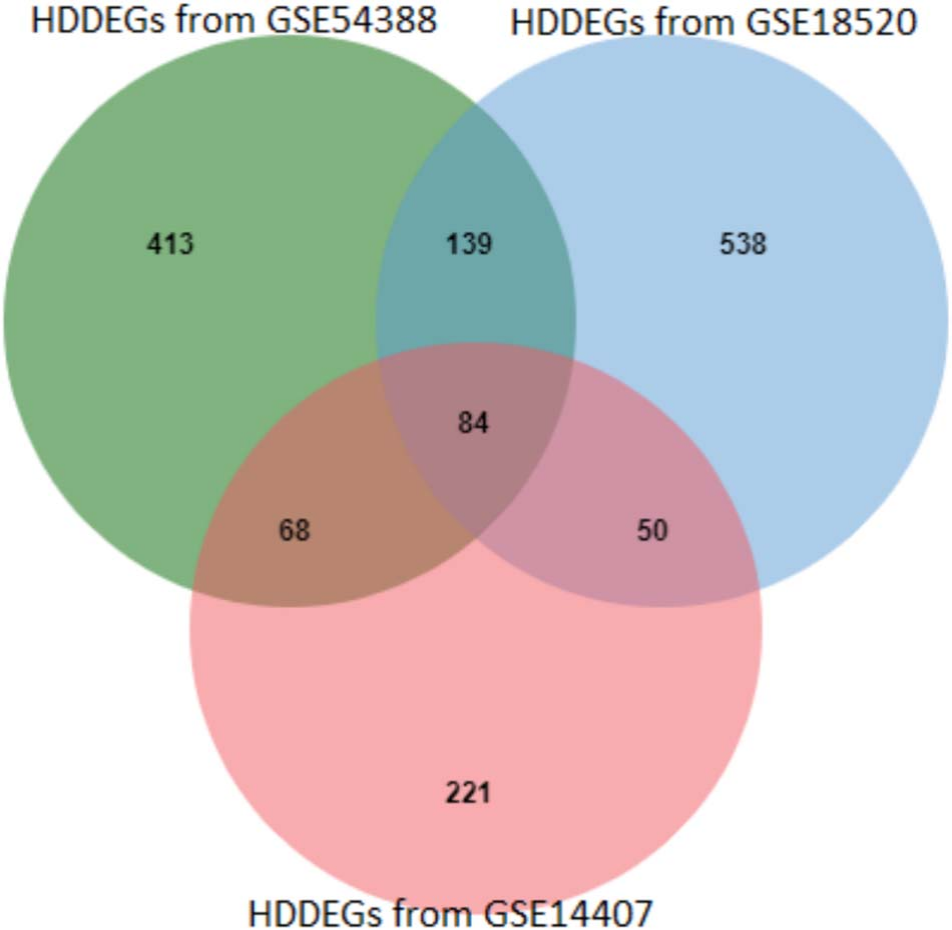
Identification of shared common HDDEGs among the microarray datasets GSE14407, GSE54388, and GSE18520 through integrative analysis.

Additionally, we identified **84 common HDDEGs** across the three datasets (GSE14407, GSE54388, and GSE18520), as shown in Fig. 4.

#### Protein–protein interaction network and central hub gene identification

**Figure 5:**
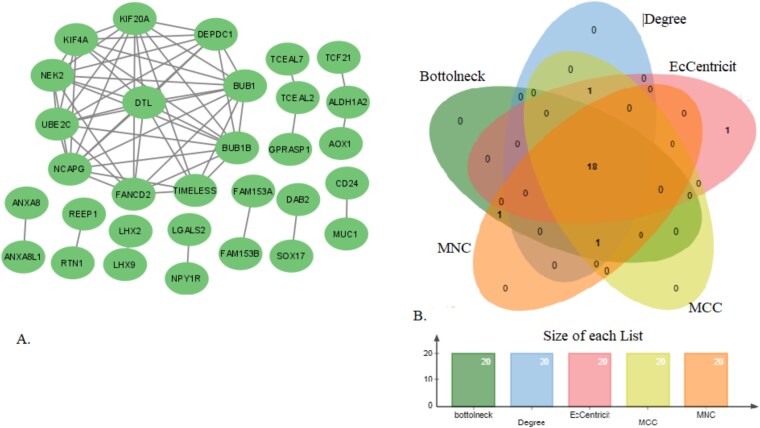
PPI network construction (A) and central hub genes identification (B).

A PPI network with 51 nodes as well as 144 edges was created for the common DEDGs using the STRING database, and it was displayed in Cytoscape (Fig. 5A). To find hub genes, we used the degree, MNC, MCC, EcCentricity, and bottleneck cytoHubba algorithms. The best 20 genes from each method were chosen. A Venn diagram (Fig. 5B) identified 18 common hub genes across all five algorithms. These hub genes included KIF20A, BUB1, NCAPG, BUB1B, DTL, UBE2C, NEK2, FANCD2, DEPDC1, KIF4A, TIMELESS, LGALS2, ANXA8, NPY1R, ANXA8L1, LHX2, ALDH1A2, and LHX9, which were considered key candidates for further analysis.

#### Identification of hub modules and associated genes

**Figure 6:**
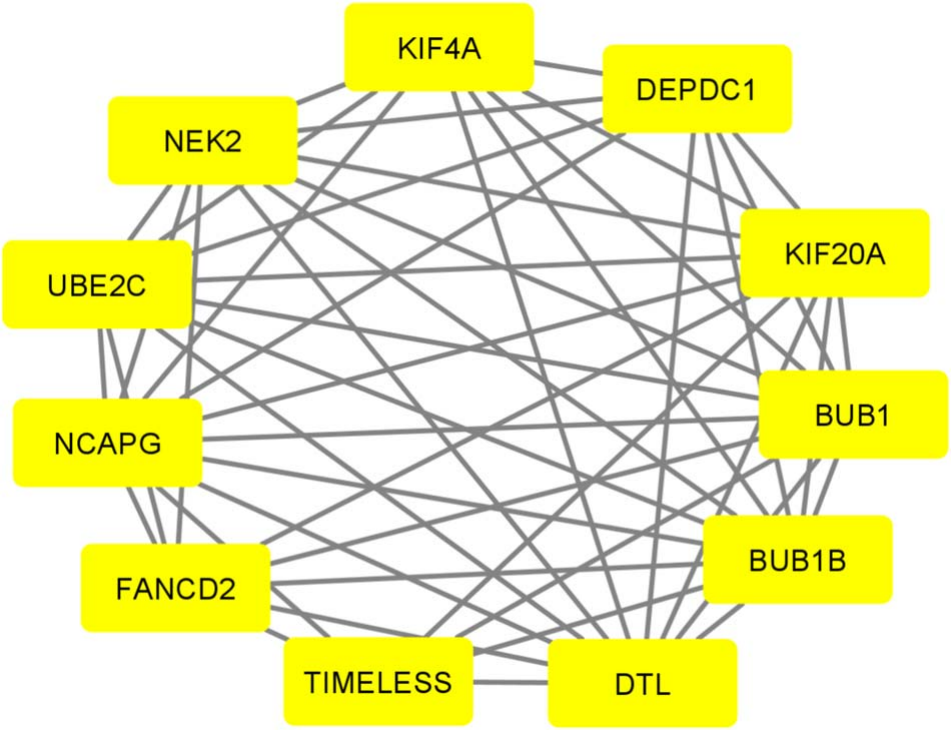
PPI network of module 1 with 11 nodes and 49 edges, which was generated by Cytoscape 3.9.s.

We conducted module analysis using MCODE on the PPI network to pinpoint key clusters. The analysis revealed three distinct modules, each exhibiting MCODE scores between 3 and 6. Among these, we identified Module 1 as the most significant, featuring 11 nodes and 49 edges, with an impressive MCODE score of 6 (see Fig. 6). The 11 genes corresponding to this prominent hub module included FANCD2, UBE2C, BUB1B, NEK2, BUB1, KIF4A, DTL, NCAPG, KIF20A, TIMELESS, and DEPDC1. These genes were recognized as the primary interconnected components of the module, highlighting their potential importance in the underlying biological processes (BPs).

#### Identification of key meta-hub genes from ovarian cancer studies

We reviewed several studies focused on OC-related gene identification to identify SMHGs in OC. The hub genes identified in various studies were compiled into a comprehensive metadata, forming a robust list of significant genes associated with OC research (see Table 2). This included 6 genes by Zhao *et al.* [8], 22 by Su *et al.* [9], 7 by Su *et al.* [10], 4 by Li *et al.* [11], and 6 by Zhou *et al.* [12]. Additional contributions included 10 genes identified by Yang *et al.* [13], 9 by Qin *et al.* [14], 7 by Liu *et al.* [15], 20 by Feng *et al.* [16], and 8 by Zhang *et al.* [17]. Similarly, 12 were noted by Li *et al.* [18], 6 by Shen *et al.* [19], 9 by Liu *et al.* [20], 3 by Yu *et al.* [21], 9 by Meng *et al.* [22], and 5 by Li *et al.* [23]. Furthermore, Tang *et al.* [24] contributed 4, Zhang *et al.* [25] 14, Meng *et al.* [26] 7, Siavoshi *et al.* [27] 7, Honar *et al.* [28] 10, and Nomiri *et al.* [29] 10. Other notable inclusions were 3 genes from Tan *et al.* [30], 4 from Chen *et al.* [31], 8 from Yuan *et al.* [32], 5 from Lai *et al.* [33], and 19 from Gur *et al.* [34]. Additionally, Wei *et al.* [35] contributed 6, Wu *et al.* [36] 4, Kasavi *et al.* [37] 6, Gui *et al.* [38] 4, Wang *et al.* [39] 3, Shao *et al.* [40] 3, and Wang *et al.* [41] 3. Finally, contributions included 5 by Moes *et al.* [42], 4 by Ma *et al.* [43], 2 by Li *et al.* [44], 3 by Li *et al.* [47], 2 by Cui *et al.* [45], and 4 by Hossain *et al.* [46].

**Table 2:** Reviewed significant hub genes from different studies

**Number of genes**	**Gene list**	**Citation**
6	AURKA, BUB1B, CENPF, KIF11, KIF23, TOP2A	Zhao *et al.* [8]
22	BUB1B, CDKN3, CENPE, CKS1B, HMMR, KIF11, KIF15, KIF18B, MCM2, MKI67, NCAPD2, RACGAP1, RAD51AP1, SPC25, NDC80, PBK, STIL, TPX2, UBE2C	Su *et al.* [9]
7	LY9, CD5, CXCL9, IL2RG, SLAMF1, SLAMF6, SLAMF7	Su *et al.* [10]
4	COL4A1, SDC1, CDKN2A, TOP2A	Li *et al.* [11]
6	CCND1, CCNE1, CXCL10, ERBB4, LPAR3, SST	Zhou *et al.* [12]
10	KIF4A, CDC20, CCNB2, TOP2A, RRM2, TYMS, KIF11, BIRC5, BUB1B, FOXM1	Yang *et al.* [13]
9	CCNB1, CDK1, BUB1, CDC20, CCNA2, BUB1B, AURKA, RRM2, TTK	Qin *et al.* [14]
7	CCNA2, CDK1, CCND1, FGF2, IGF1, BCL2, VEGFA	Liu *et al.* [15]
20	BUB1, BUB1B, CCNB1, CDCA5, CENPF, DEPDC1, ECT2, FAM83D, FOXM1, HMMR, KIF11, NCAPG, RAD51AP1, TTK, UBE2C	Feng *et al.* [16]
8	NDC80, PDGFD, SNCA, AGR2, PSAT1, EFEMP1, HOXD8, HMGA1	Zhang *et al.* [17]
12	CDK1, TOP2A, CDC20, CCNB2, BIRC5, UBE2C, BUB1, NCAPG, RRM2, KIF2C, CENPA, MELK	Li *et al.* [18]
6	DTL, DLGAP5, KIF15, NUSAP1, RRM2, TOP2A	Shen *et al.* [19]
9	SFRP1, PSAT1, BUB1B, FOLR1, ABCB1, PDE8B, INAVA, BUB1, TMEM139	Liu *et al.* [20]
3	ALDH1A2, CLDN4, GPR37	Yu *et al.* [21]
9	WT1, IGFBP1, FBN1, SERPINA1, MFAP4, LTBP4, IGF2, IGF1, ITGB6	Meng *et al.* [22]
5	CDK1, CCNB1, AURKA, CDC20, CCNA2	Li *et al.* [23]
4	TTK, BUB1B, NUSAP1, ZWINT	Tang *et al.* [24]
14	ADH1B, CDH11, VGLL3, KIT, GF2, GABRB2, DENND2A, AOX1, ALDH1A1, ADCY8, FOXL2, MUM1L1, ACPP, ALPPL2, AQP5, APOF, BST2	Zhang *et al.* [25]
7	BUB1, CDC20, CCNB2, DLGAP5, KIF4A, NEK2, NUSAP1	Meng *et al.* [26]
7	UBC, FN1, SPP1, ACTB, GAPDH, JUN, RPL13A	Siavoshi *et al.* [27]
10	KIAA0101, RAD51AP1, FAM83D, CEP55, PRC1, CKS2, CDCA5, NUSAP1, ECT2, TRIP13	Honar *et al.* [28]
10	ADORA1, ANO9, CD24P4, CLDN3, CLDN7, ELF3, KLHL14, PRSS8, RASAL1, RIPK4, SERINC2, WNT7A	Nomiri *et al.* [29]
3	GOGA8B, LRRC26, CCDC114	Tan *et al.* [30]
4	COL6A3, CRISPLD2, FBN1, SERPINF1	Chen *et al.* [31]
8	EDIL3, NRAS, HAPLN1, PROCR, CD53, CDKN2A, IGF1R, ROBO2	Yuan *et al.* [32]
5	TTLL10, KRT8, SGCG, OSBPL10, ACOXL	Lai *et al.* [33]
19	ACTB, AKT1, ALB, CTNNB1, EGFR, EP300, ESR1, FN1, GAPDH, HSPA4, IL6, JUN, MYC, PTEN, RPS27A, SRC, TNF, TP53, UBC	Gur *et al.* [34]
6	CCNB1, TOP2A, NUSAP1, NCAPG, KIF20A, DLGAP5	Wei *et al.* [35]
4	FANCD2, PKD2, TBRG1, DOCK5	Wu *et al.* [36]
6	CDT1, CNIH4, CRLS1, LIMCH1, POC1A, SNX13	Kasavi *et al.* [37]
4	CDC45, CDCA5, KIF4A, ESPL1	Gui *et al.* [38]
3	ALB, APOB, SERPINA1	Wang *et al.* [39]
3	EZR, HSPG2, SLC9A1	Shao *et al.* [40]
3	C9orf16, COX5B, ACTB	Wang *et al.* [41]
5	FANCD2, BRIP1, BRCA1, BRCA2, FANCF	Moes *et al.* [42]
4	UBE2C, CDC20, PTTG1, AURKA	Ma *et al.* [43]
2	UBE2C, CDK1	Li *et al.* [44]
3	CCNF, KIF20A, FOXM1	Li *et al.* [47]
2	DTL, PDCD4	Cui *et al.* [45]
5	TLR4, BSCL2, CDH1, ERBB2, SCGB2A1	Hossain *et al.* [46]

This extensive dataset, supported by prior studies, underscores the robust identification of critical biomarkers and therapeutic targets in OC. This wide range of genes collected ensures a robust and diverse dataset for further analysis. These MHGs were compiled, and their frequency of occurrence across studies was analyzed to identify significant genes that consistently appeared in multiple research findings, providing a strong basis for their role as key candidate genes in OC.

#### Analyzing the enrichment of shared highly discriminative differentially expressed genes of ovarian cancer

**Figure 7:**
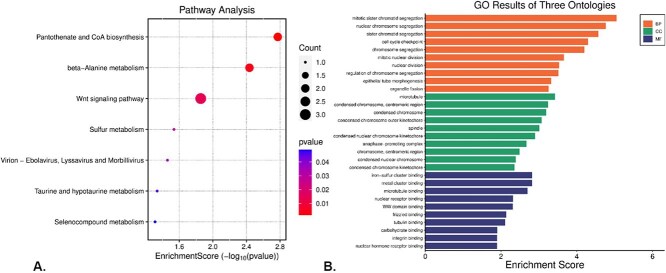
Enrichment analysis of shared HDDEGs in OC. (A) KEGG pathway analysis reveals key biological pathways associated with the shared genes. (B) GO ontology analysis highlights the BPs, cellular components (CCs), and molecular functions (MFs)enriched among the shared HDDEGs.

Pathway and GO analyses were conducted on the identified common significant HDDEGs to unveil shared biological pathways and functional categories. Using the ClusterProfiler R package [54], we identified significant KEGG pathways such as Pantothenate and CoA biosynthesis, beta-Alanine metabolism, Wnt signaling pathway, Sulfur metabolism, Taurine and hypotaurine metabolism, and Selenocompound metabolism. Additionally, viral pathways like Ebolavirus, Lyssavirus, and Morbillivirus were enriched (see Fig. 7A).

**Figure 8:**
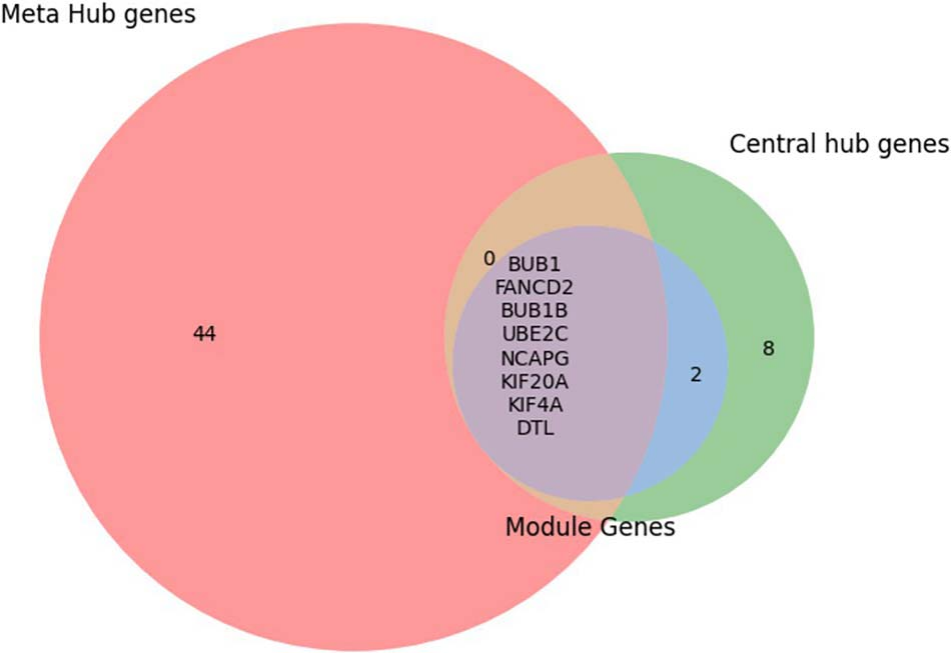
Identification of key candidate genes of OC from central hub genes, hub module genes, and SMHGs.

For GO analysis, the top 10 enriched terms for BPs, CCs, and MFs were identified (see Fig. 7B).

#### Identification of key candidate genes

Using five different analytical approaches—connectivity degree, MNC, MCC, EcCentricity, and bottleneck—we identified 18 central hub genes that play a crucial role in OC. Additionally, 11 hub module genes were discovered from potential hub modules, while 54 MHGs were identified through meta-hub analysis. By overlapping these three gene sets through a Venn diagram, eight key genes were highlighted: FANCD2, BUB1, BUB1B, KIF4A, DTL, NCAPG, KIF20A, and UBE2C. These genes stood out as significant because they can effectively distinguish between OC and healthy tissues. As shown in Fig. 8, these eight genes are likely to be important markers in OC research, potentially offering new insights into disease progression and treatment strategies.

#### Validation of key candidate genes

The discriminative power of eight key candidate genes (*FANCD2*, *NEK2*, *BUB1*, *KIF4A*, *DLT*, *NCAPG*, *KIF20A*, and *UBE2C*) was evaluated using ROC curve analysis, and the AUC values were computed across three datasets: **GSE18520** (training dataset), **GSE38666**, and **TCGA-OC** (independent test datasets). These analyses aim to assess the precision and robustness of these genes in distinguishing OC patients from healthy controls.

**Figure 9:**
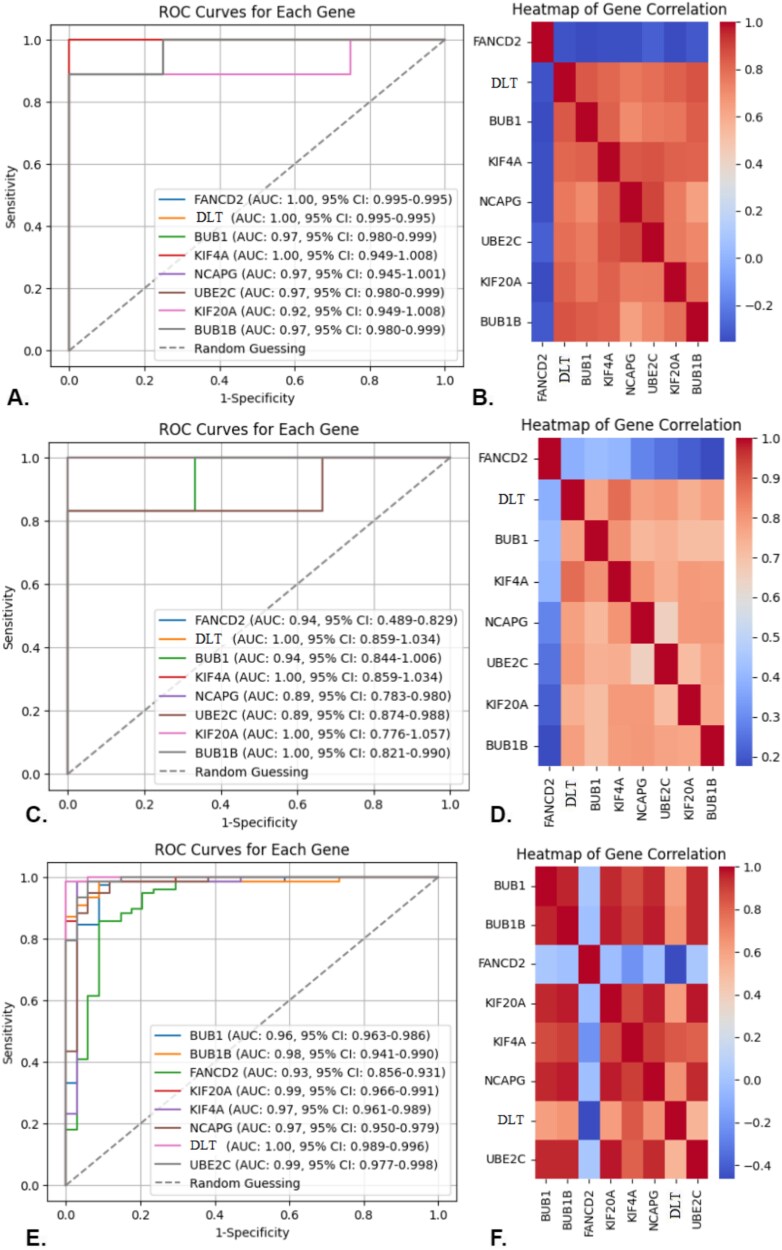
Evaluation of the eight primary candidate genes via AUC and heatmap analysis: (A) and (B) correspond to the GSE18520 training dataset; (C) and (D) correspond to the GSE38666 independent test dataset; and (E) and (F) correspond to the TCGA-OC independent test dataset.

For the **GSE18520 training dataset** (Fig. 9A and B):

- *FANCD2*, *DLT*, and *KIF4A* exhibited perfect discriminative power, each with an AUC of 1.00 (95% CI: 0.995–1.000), indicating strong classification ability for OC.
- Other genes also performed well: *BUB1* (AUC: 0.97, 95% CI: 0.980–0.999), *NCAPG* (AUC: 0.97, 95% CI: 0.945–1.001), *UBE2C* (AUC: 0.97, 95% CI: 0.980–0.999), *KIF20A* (AUC: 0.92, 95% CI: 0.949–1.008), and *BUB1B* (AUC: 0.97, 95% CI: 0.980–0.999), demonstrating significant predictive capability.

The heatmap analysis (Fig. 9B) showed high correlations among these genes, suggesting their co-expression in OC patients.

In the **GSE38666 independent test dataset** (Fig. 9C and D):

- *DLT* and *KIF4A* retained their perfect discriminative power, both with an AUC of 1.00 (95% CI: 0.859–1.034), highlighting their reliability across datasets.
- *FANCD2* (AUC: 0.94, 95% CI: 0.489–0.829), *BUB1* (AUC: 0.94, 95% CI: 0.844–1.006), *NCAPG* (AUC: 0.89, 95% CI: 0.783–0.980), *UBE2C* (AUC: 0.89, 95% CI: 0.874–0.988), *KIF20A* (AUC: 1.00, 95% CI: 0.776–1.057), and *BUB1B* (AUC: 1.00, 95% CI: 0.821–0.990) also showed strong performance, validating their relevance.

The heatmap (Fig. 9D) demonstrated significant co-expression and correlation between these genes, suggesting their shared role in OC pathogenesis.

For the **TCGA-OC independent test dataset** (Fig. 9E and F):

- *BUB1* (AUC: 0.96, 95% CI: 0.963–0.986), *BUB1B* (AUC: 0.98, 95% CI: 0.941–0.999), *FANCD2* (AUC: 0.93, 95% CI: 0.856–0.931), *KIF20A* (AUC: 0.99, 95% CI: 0.966–0.991), *KIF4A* (AUC: 0.97, 95% CI: 0.961–0.989), *NCAPG* (AUC: 0.97, 95% CI: 0.950–0.979), *DLT* (AUC: 1.00, 95% CI: 0.989–0.996), and *UBE2C* (AUC: 0.99, 95% CI: 0.977–0.998) displayed excellent predictive power.

The corresponding heatmap (Fig. 9F) further confirmed the tight correlation between these key genes, reinforcing their importance in distinguishing OC patients from healthy individuals.

These results demonstrate that the eight key genes possess strong discriminative capabilities, with AUC values exceeding 0.90 across the three datasets. This robust performance across independent datasets highlights their potential as reliable biomarkers for OC detection, supporting their use in clinical diagnostic models. The heatmap analyses complement these findings by showing consistent co-expression patterns, suggesting these genes play coordinated roles in OC pathogenesis.

#### Survival analysis of key candidate genes of ovarian cancer

**Figure 10:**
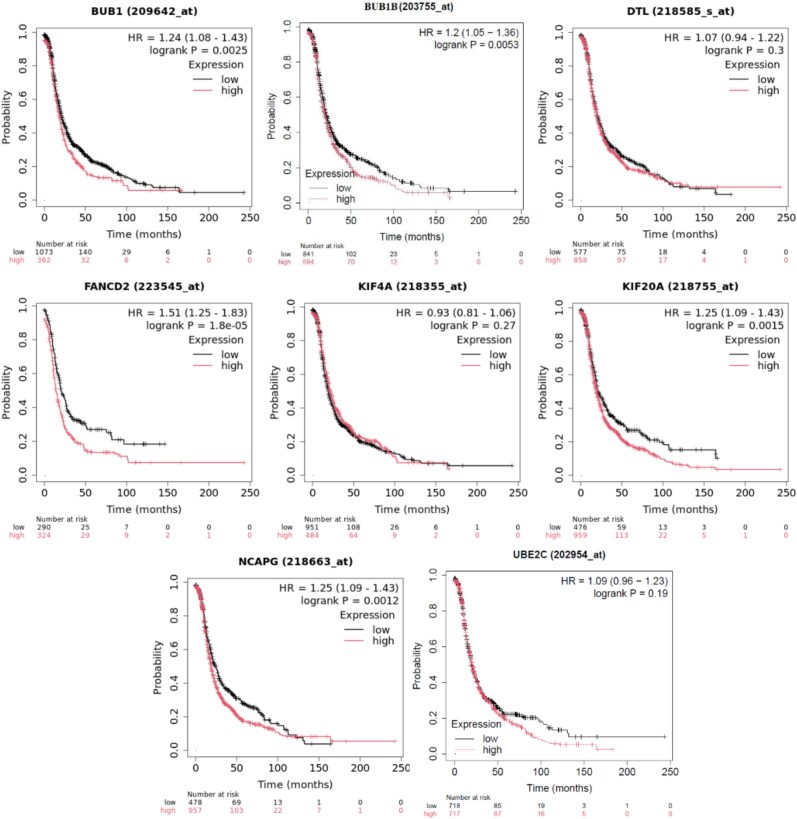
Survival curve of eight Significant genes (FANCD2, BUB1, BUB1B, KIF4A, DTL, NCAPG, KIF20A, and UBE2C).

In the survival analysis conducted on the eight significant genes—FANCD2, BUB1, BUB1B, KIF4A, DTL, NCAPG, DEPDC1, and UBE2C—only five genes (FANCD2, BUB1, BUB1B, NCAPG, and KIF20A) demonstrated significant associations with patient survival based on $P$-values $<.05$ (see Fig. 10). Specifically, FANCD2 exhibited a hazard ratio (HR) of 1.51, BUB1 showed an HR of 1.24, BUB1B had an HR of 1.2, NCAPG showed an HR of 1.25, and KIF20A demonstrated an HR of 1.25, indicating that higher expression of these genes may be associated with poorer patient outcomes. The remaining genes (KIF4A, DTL, and UBE2C) did not reach statistical significance in this analysis.

#### Weighted gene co-expression network analysis analysis of common highly discriminative differentially expressed genes

**Figure 11:**
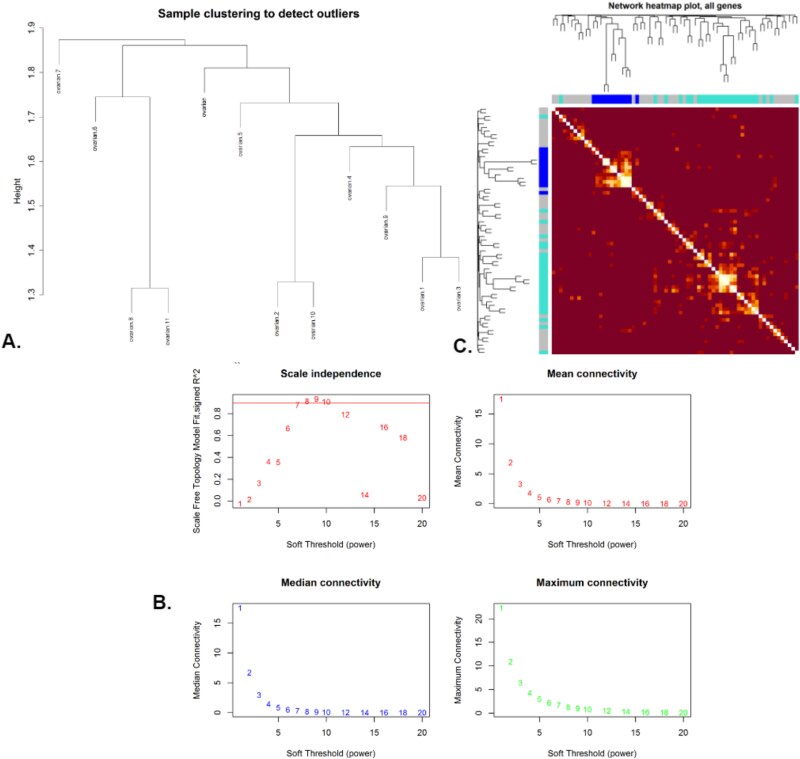
WCGNA analysis of Shared genes using the dataset GSE14407. (A) Cluster dendrogram of the samples showing hierarchical clustering and detection of outliers. (B) Co-expression network plot illustrating the relationships among genes based on topological overlap. (C) Network heatmap of all genes representing the strength of co-expression across the dataset.

We identified 84 common HDDEGs across the three datasets: GSE14407, GSE54388, and GSE18520. We extracted these genes’ normalized expression values from the GSE14407 OC dataset and utilized them to construct gene co-expression networks using WGCNA analysis. After clustering the samples to detect outliers and selecting an optimal soft-thresholding power to achieve a scale-free network, we identified modules of co-expressed genes and related them to clinical traits. Ultimately, we found 68 genes that formed meaningful clusters. Notably, eight key genes (FANCD2, NEK2, BUB1, KIF4A, DTL, NCAPG, KIF20A, and UBE2C) were identified within the same module, suggesting shared regulatory mechanisms or pathways. Visualizations included sample clustering dendrograms, scale independence and connectivity plots, network heatmaps, module–trait relationship heatmaps, eigengene network heatmaps, and a network plot highlighting the key genes’ interconnectedness, as shown in Fig. 11.

## Discussion

This study used the “limma” program to find DEGs in three OC datasets: GSE14407, GSE54388, and GSE18520. We found 1486 DEGs from GSE14407, including 661 upregulated and 825 downregulated genes. We found 1415 DEGs for GSE54388, including 695 upregulated and 720 downregulated genes. Last, out of the 1143 DEGs found in GSE18520, 629 were upregulated, and 514 were downregulated. Figure 2 displays the findings from these datasets.

To further refine the gene sets, we implemented SVM, which allowed us to filter out HDDEGs. This analysis yielded 390 HDDEGs from GSE14407, 712 HDDEGs from GSE54388, and 811 HDDEGs from GSE18520. The volcano plots and heatmaps for these DEGs are presented in Fig. 2, illustrating the distribution of upregulated and downregulated genes across the datasets. We found 84 overlapping HDDEGs among the datasets (Fig. 4), which were subjected to enrichment analysis to explore their roles in OC development and molecular mechanisms (Table 1). A PPI network was constructed with these HDDEGs. Using five analytical approaches—connectivity degree, MNC, MCC, EcCentricity, and bottleneck—we identified 18 central hub genes critical to OC. Additionally, 11 hub module genes and 54 MHGs were identified. Lapping these sets through a Venn diagram highlighted eight key genes (FANCD2, BUB1, BUB1B, KIF4A, DTL, NCAPG, KIF20A, and UBE2C). These genes effectively distinguish OC from healthy tissues and hold promise as biomarkers and therapeutic targets, offering valuable insights into OC progression and treatment. These genes demonstrate strong diagnostic potential and provide insights into therapeutic strategies.

For instance, recent studies have emphasized the pivotal role of mitotic kinases BUB1 and BUB1B in OC prognosis and treatment outcomes. High expression of BUB1B is correlated with poor overall survival in epithelial OC, chemotherapy resistance, and unfavorable progression-free survival in early-stage cases [55–57]. These insights highlight the potential of BUB1 and BUB1B as significant prognostic biomarkers and therapeutic targets in OC management.

Different existing studies also supported our findings BUB1 [14, 16, 18, 20, 26] and BUB1B [8, 9, 14, 16, 20, 24] as potential biomarkers of OC.

Similarly, DTL has emerged as a critical biomarker, with studies showing its significant overexpression in OC tissues compared to normal ovarian tissues [19]. Furthermore, its expression correlates with poor prognosis and adverse clinical outcomes, as revealed through pan-cancer analyses and OC-specific investigations [45, 58]. These findings suggest that DTL could serve as both a prognostic marker and a therapeutic target.

Likewise, NCAPG plays a crucial role in OC progression. It is significantly overexpressed in OC tissues and cell lines, which correlates with poor survival and aggressive tumor behavior [59, 60]. Moreover, its knockdown has been shown to inhibit cancer cell proliferation, invasion, and migration while promoting apoptosis and enhancing cisplatin sensitivity. This effect appears to be mediated through the p38 MAPK signaling pathway [60]. Thus, NCAPG stands out as a promising target for therapeutic intervention [16, 18].

Furthermore, adverse clinical results as well as the progression of OC have been linked to KIF20A, a crucial mitotic kinesin. Poor survival rates, lymph node metastases, and advanced illness stages are all correlated with its overexpression [47, 61]. Additionally, it encourages OC cells to invade, metastasize, and resist chemotherapy, highlighting its significance as a crucial prognostic and therapeutic target [35, 62]. Furthermore, in patients receiving platinum/taxol treatment, KIF20A overexpression is associated with a poorer prognosis [62]. Our results in line with previous studies imply that KIF20A may be a viable biomarker for OC [35, 47].

FANCD2, a crucial protein in the Fanconi anemia/BRCA pathway, is one of these and has a complex relationship with OC. Many ovarian cancer patients (OCPs) have reduced FANCD2 expression [63], whereas overexpression of the protein is associated with a poor prognosis, especially in patients undergoing taxane-platinum therapies [42]. Remarkably, better survival in OCP is correlated with FANCD2’s cytoplasmic location [63]. Platinum resistance in high-grade serous OC may also result from FANCD2 upregulation, which may be mediated by mTOR [64]. Cytoplasmic FANCD2 is involved in cell migration in addition to DNA repair [64]. Our findings support earlier research [36, 42], highlighting the potential of FANCD2 as well as other proteins as OC biomarkers and treatment targets.

Another interesting biomarker and treatment target for OC is KIF4A, a kinesin family member. According to studies, it is overexpressed in OC tissues and cell lines, which is associated with a poor prognosis. KIF4A knockdown induces apoptosis and inhibits OC cell migration, proliferation, and colony formation [65]. Additional research backs up its link to the course of the disease and its possibility for treatment [13, 26, 38]. These results demonstrate the broader potential of members of the kinesin family as targets for cancer diagnosis and treatment, especially OC.

Similarly ubiquitin-conjugating enzyme UBE2C plays a crucial role in the pathophysiology of OC. In high-grade serous OC, it is additionally overexpressed but also hypomethylated, which is associated with later stages and a bad prognosis [44]. Its depletion also restores cisplatin resistance, causes apoptosis, and inhibits the growth of cancer cells. According to earlier research, UBE2C has thus become a crucial biomarker for OC [9, 43]. Crucially, AUC analysis was used to evaluate the predictive value of these eight essential candidate genes—FANCD2, BUB1, BUB1B, KIF4A, DTL, NCAPG, KIF20A, and UBE2C—using two separate test datasets. Additionally, a survival analysis showed a strong correlation between them and the onset and spread of OC. These results highlight the significance of these genes in comprehending the pathophysiology of OC and may help with diagnosis and prognosis. When combined, they offer a strong basis for improving OC treatment approaches and diagnosis accuracy. These findings collectively demonstrate the significance of these key genes in OC progression and prognosis. Their strong associations with survival outcomes and treatment resistance suggest they could serve as valuable biomarkers and potential therapeutic targets, warranting further experimental validation and clinical investigation. Although this study provides valuable insights into OC’s molecular mechanisms, it has limitations. These include reliance on publicly available transcriptomic data, lack of experimental validation such as qPCR, and potential biases due to retrospective analysis and dependence on public databases.

## Conclusion

In conclusion, our study identifies key candidate genes potentially critical to OC progression and prognosis. Through the analysis of DEGs from three datasets (GSE14407, GSE54388, and GSE18520) and validation using independent test datasets, we highlight FANCD2, BUB1B, KIF4A, DTL, NCAPG, KIF20A, DEPDC1, and UBE2C as potential biomarkers. The consistent findings across datasets support these genes’ significance in OC. Furthermore, our study emphasizes the role of gene expression and regulatory mechanisms in understanding cancer pathogenesis. These findings offer a foundation for future functional studies and may provide a basis for developing novel diagnostic and therapeutic strategies for OC.

**Key Points**

- **High-accuracy gene selection:** Integrative analysis using machine learning (support vector machine) achieved 90% accuracy in filtering differentially expressed genes and identified 84 highly discriminative differentially expressed genes across three datasets.
- **Network and module insights:** WGCNA analysis grouped the eight key genes into a single cluster, and PPI analysis identified central hub genes pivotal to ovarian cancer.
- **Meta-hub gene identification:** A meta-analysis highlighted 54 meta-hub genes (MHGs) from prior studies, underscoring their significant role in cancer biology.
- **Key gene identification and validation:** Eight shared key genes (*FANCD2*, *BUB1B*, *BUB1*, *KIF4A*, *DTL*, *NCAPG*, *KIF20A*, and *UBE2C*) were identified by intersecting central hub, hub module, and MHGs and validated through AUC and survival analyses.
- **Comprehensive workflow application:** The study integrated bioinformatics and machine learning techniques, demonstrating a robust framework for biomarker discovery and prognostic analysis in ovarian cancer research.

## Data Availability

We obtained our datasets for ovarian cancer from publicly available repositories. Specifically, we utilized three microarray datasets from the NCBI GEO database with the accession numbers GSE14407, GSE54388, and GSE18520. Additionally, we used independent datasets for validation, including GSE38666 and mRNAseq data from The Cancer Genome Atlas for Ovarian Cancer (TCGA-OC) available through the TCGA genome data analysis centre (http://gdac.broadinstitute.org/). We also included normal ovarian tissue data comprising 180 patient samples obtained from the GTEx project, accessible at https://www.gtexportal.org/home/. Our study’s code and processed data can be made available upon request for further research or verification purposes.
